# Ohio State Medical Society

**Published:** 1870-09

**Authors:** Wm. B. Davis

**Affiliations:** Cincinnati, Ohio


ARTICLE XXVIII.

### OHIO STATE MEDICAL SOCIETY.

### VACCINAL SYPHILIS.

PART OF A REPORT ON VACCINATION.

BY WM. B. DAVIS, M.D., of Cincinnati, Ohio.

Over seventy years have elapsed since the introduction of vaccination by Jenner. The majority of the population of the civilized world have been vaccinated. If the vaccine lymph will convey constitutional diseases, other than vaccinia, its wide-spread use, during these seventy years, will show a large increase of constitutional affections.

A careful examination of the record for this period does not show that there has been such an increase. On the contrary, statistics, according to Farr, Greenhow, and others, prove that scrofula and other constitutional diseases have diminished, and that the average duration of life has been extended, since the introduction of vaccination.

The *British and Foreign Medico-Chirurgical Review*, for Jan. 1869, makes the following statement, page 126: "It can not be disputed that the average tenure of life has been lengthened by vaccination, and that a most horrible and disgusting disease (which, where it did not kill, often rendered its victim blind or deaf, and generally hideous for the remainder of life) has been well-nigh exterminated.

Niemeyer of the University of Tubingen, in his recent work Practical Medicine says on page 553, Vol. II: "All objections to vaccination, even if well founded, would to have to give way to the facts proved by statistics, that in the last century one-tenth of the population died of small-pox (about 400,000 people dying of the disease every year in Europe), another one-tenth were disfigured by the disease; and that since the introduction of vaccination, the general mortality is less, and that of small-pox is reduced to a minimum."

There are certain cutaneous eruptions which follow the operation of vaccination, just as they may follow teething, or any other slight constitutional disturbance. Mr. Padget pointed out this fact twelve years since, when he wrote that "multitudes of children are naturally predisposed to the commoner eruptions, and that vaccination is only one of the trifling constitutional irritations, which might have precipitated the first appearance of such eruptive diseases." And more recently Niemeyer, in his Practical Medicine, says: "We know that blisters and other irritants to the skin, not only induce inflammation at the point of application, but also increase the predisposition to other cutaneous affections. Many children who have never previously suffered from any exanthema, are affected for months with moist eczema of the face, after having their ears pierced, as well as after vaccination.

Vaccine lymph, when long exposed to air and moisture, will undergo the same putrefactive changes that any other animal matter does when similarly exposed; and if inserted in the arm of a subject, will produce the same effects that any other putrid matter will produce. When erysipelas or pyæmia follow the use of decomposed lymph, the vaccine is not at fault, but the vaccinator, who has been criminally careless, or criminally ignorant.

The advocates of transmission, however, do not press these minor points, and seem, indeed, to have abandoned their claim for the transmission of all constitutional diseases but that of Syphilis. Upon this one disease they rally their full strength. I shall, therefore, now invite your attention to the consideration of the possibility of engrafting syphilis by means of Jennerian vaccination.

It is a fact worthy of notice, that a large majority of all the reported cases of vaccinal syphilis, have occurred in France and

Italy. In England, where vaccination is more systematically pursued, and where the official inspection, to which it is subjected, would render the escape of any abnormal development impossible; there are but a very few cases reported.

Whether or not, syphilis, independent of vaccination, is more prevalent in France and Italy than other countries, is a consideration of some moment in its bearing on this question. An affirmative answer might suggest a satisfactory solution of vaccinal-syphilis, so far as these two countries are concerned, without implicating the vaccine lymph.

According to Padget, the transmission of syphilis by means of vaccination, is opposed by pathological considerations. Such pathologists as Rokitansky, Skoda, and Hebra maintain that the insertion of a matter containing the virus of two different contagious affections, is followed by the production of but one disease. That is, if the matter used be a mixture of syphilitic and vaccine virus, either distinct syphilis or distinct vaccinia is the result; not both of them.

It is opposed by direct experiment. Lymph intentionally taken from vaccine vesicles on syphilitic subjects, produced only vaccination.

M. Delzenne recently reported in the Imperial Academy of Medicine, Paris, that in 1865, he had inoculated himself twice with vaccine from syphilitic subjects, and the result was negative. In September, 1866, he vaccinated a syphilitic patient, 21 years old, "who had numerous ulcerated, hypertrophied papules in vulva and perineum, a general papular eruption (syph) over body and mucous patches in the mouth and throat. On the eighth day he vaccinated himself in four places with the virus from this patient, and the result was negative. He also, at the same time, vaccinated seven females (free from syphilis) with the same lymph. The effect so far as syphilis was concerned was entirely negative—four responded perfectly to the vaccine.

M. Bourguel reported twenty cases, whom he had vaccinated with lymph from the arms of two syphilitic subjects, and the result was negative for syphilis. M. Guerin reported fifty-five experiments, combining all the characteristics of scientific experiment, which responded negatively to the artificial production of vaccinal syphilis.

These confirm the experiments previously made by Cullerier, Taupin, Heim, and others.

If, however, the matter from a chancre be accidentally or intentionally inserted in the arm of a patient, it will produce syphilis, but the lymph from a vaccine vesicle on the arm of a syphilitic subject, will only produce vaccinia, just as the lymph from a vaccine vesicle on a patient who is broken out with the small-pox (the vaccination having been performed too late for protection), will only produce vaccination, while the pustules by its side will produce small-pox.

It is opposed by the experience of the greatest vaccinators and syphilographers. West, with an experience of 26,000 vaccinations; Marson, with 40,000; Sir W. Jenner, with 13,000; Seaton, with 50,000; Tomkins, with 50,000; Perkins, with 40,-000; Loines, with 200,000; Clendenin, with 16,000, and many others who might be named, have never met with a case of vaccinal syphilis.

The following question was addressed by Mr. Simon, in 1856, to the most distinguished medical gentlemen of Germany, France, and Great Britain: "Have you any reason to believe or suspect that lymph from a true Jennerian vesicle has ever been a vehicle of syphilitic, scrofulous, or other constitutional infection to the vaccinated person?" Nearly six hundred replies were received, and they were almost unanimously in the negative. Among whom were Padget, West, Marson, Tomkins, Acton, Lee, Parker, Chomel, Bright, Watson, and Brodie.

A large proportion of the reported cases of vaccinal syphilis rests upon insufficient or defective evidence, and the remainder may be reasonably accounted for, without compromising the vaccine, on the grounds (1) of the influence of prevailing diseases; (2) a cachetic diathesis, and (3) latent constitutional syphilis.

Vaccinal-syphilis had but few advocates, either in or out of the profession, until the so-called epidemics of "Rivalta," in 1861, and subsequently of "Auray" (provinces of Italy and

France) made their appearance. These epidemics aroused the attention of the profession, and led to a thorough investigation of the causes which produced them.

Concerning the "Rivalta" cases, Ricord says: "For our part, an attentive perusal of the documents of the case, has led us to the same conclusion as Dr. Abertetti, who exonerates from all blame the vaccination in question." With reference to the Auray epidemic, Dr. Anstie, in the *Practitioner*, of November, 1869, says: "Upon the whole evidence we say, decidedly, that it is not proved that the epidemic of Auray was an epidemic of syphilis at all, far less that it was an epidemic of vaccine syphilis."

Dr. Seaton calls attention in his *Handbook* to the fact, that long before vaccination was heard of, Astruc recorded epidemics of syphilis, which were full as disastrous and as unaccountable as that of Rivalta.

In the recent discussion in the French Academy, M. Depaul, Director of Vaccination, reported a large number of cases of vaccinal syphilis, as having occurred in the arondissements of Lorient and Vannes.

M. Fouquet stated that there were other vaccinations in those districts with accidents analogous to those reported by M. Depaul, *viz.:* highly-inflamed pustules, large deep ulcerations, abundant eruptions, etc., but that these accidents had not an impure origin, and presented nothing suspicious, although the characters noted by him had the greatest analogy with those reported syphilitic by M. Depaul. M. Guerin asserted that the explanation of those cases of M. Depaul, was to be found in a general erysipelatous diathesis prevailing in those departments. He called attention to the fact that any local irritation would develop a latent constitutional taint, and said that *vaccination would not be a port of entry for syphilis, but a means of exit.* In support of the assertion that prevailing diseases modify vaccinations and produce suspicious sores, he referred to the work of M. Lalagade, Director of Vaccinations of Tarn. On May 25th, he vaccinated 95 children with lymph from a healthy child. On June 6th, a number of them were found in a sad condition, "the skin was of a deep red, with erysipelatous aspect, vaccinal pustules, very large and grayish white, black crusts all over the body, except the soles of the feet, and on the genital organs; one was covered with red patches similar to measles, another had, the day after the vaccination, large bullæ filled with serum." There was, also, an epidemic of pemphigus, complicating the inoculations. On inquiry into the public health in the neighborhood, he found erysipelas, diphtheritic anginæ, malignant pustule, wounds with sanious bases, and numerous cases of pemphigus. On April 10, 1869, he adds: "I am happy to say that to-day none of the children who were vaccinated May 25th, 1868, bear even a doubtful trace of syphilitic disease, and that none of them had undergone specific treatment.

M. Guerin concludes, on this point, by observing:

1. That vaccinal syphilis, up to this time, fails in the majority of points which would support a belief in such an origin.

2. That experiments instituted to determine the possible inoculation of vaccinal syphilis, are quite contrary to the doctrine of syphilitic vaccination.

3. That among a large number of alleged facts of vaccinal syphilis, most, if not all, belong evidently to another order of pathological influences.

And further along in the discussion, he asserts that the causes which are likely to vitiate vaccine and give it the false appearance of syphilis, are of a nature to exercise their influence equally upon human and animal vaccine. These causes, foreign to the vaccinifer, are either exterior to the subject vaccinated, or inherent in his constitutional state—both more or less susceptible of being determined, prevented, or combated.

I recently addressed a letter to Dr. J. K. Barnes, Surgeon General U. S. Army, asking him if there was any evidence on file in his office which reported the existence of any vaccinal syphilis among the 2,000,000 of soldiers who took part in the late American war. In reply, he very kindly wrote: "That quite a number of papers are on file in this office bearing on the subject of foul ulcers following vaccination, and supposed by some to be due to the contamination of the matter employed with syphilitic virus. I have made a preliminary examination of these papers, and have arrived at the conclusion that their general tenor is directly opposed to this supposition."

Prof. J. Jones, of New Orleans, has published a paper on "Spurious Vaccination," based on materials furnished by the Confederate Army. His conclusions agree with those of the Surgeon General, in exonerating the vaccine from blame, and referring the foul ulcers which attended vaccination in the army, to the fatigue, exposure, uncleanliness, insufficient and improper diet, etc., incident to a soldier's life, which resulted in a cachetic diathesis.

Wm. Clendenin, M.D., Medical Director of Hospitals, U. S. A., at Nashville, during the late war, and at the present time Health Officer of Cincinnati, has furnished me a report of his views on vaccination. His experience and opportunities for personal inspection of the ulcers, and so-called cases of vaccinal syphilis, which followed vaccination in the U. S. Army, were so great, that his views concerning them will be of value. His conclusions fully accord with those above expressed.

In reply to my inquiry, Dr. Seaton, in his letter of April 9th, writes: "I am not aware of any fresh cases of alleged introduction of syphilis by vaccination with humanized lymph, since I published my book, but curiously enough there has been discussion lately in France on some cases of syphilis in children, who had been vaccinated with animal lymph. Of course, the syphilis was a latent syphilis, and the vaccination could have nothing to do with it, except, perhaps, to hasten its evolution; but the cases are instructive, and point, in my opinion, to the explanation of all the alleged cases of vaccino-syphilitic inoculation with humanized lymph, except those in which there was downright carelessness and mixture or substitution of viruses."

This opinion of Dr. Seaton is supported by M. Guerin's terse assertion in the Academy, that "Vaccination is not a port of entry for syphilis, but a means of exit;" and by the *Medico-Chirurgical Review*, January, 1869, which says, "it is not the vaccination, but the parentage which is at fault."

I will close this section of my report by quoting the declaration of Dr. Anstie, in the *Practitioner*, of November, 1869: "*Vaccinal syphilis is a bugbear and a phantom.*"

